# A Ser252Trp Mutation in Fibroblast Growth Factor Receptor 2 (FGFR2) Mimicking Human Apert Syndrome Reveals an Essential Role for FGF Signaling in the Regulation of Endochondral Bone Formation

**DOI:** 10.1371/journal.pone.0087311

**Published:** 2014-01-28

**Authors:** Peng Chen, Li Zhang, Tujun Weng, Shichang Zhang, Shijin Sun, Mingtao Chang, Yang Li, Bo Zhang, Lianyang Zhang

**Affiliations:** 1 State Key Laboratory of Trauma, Burns and Combined Injury, Institute of Surgery Research, Daping Hospital, Third Military Medical University, Chongqing, China; 2 Neurosurgery Department, PLA 324 Hospital, Chongqing, China; Hungarian Academy of Sciences, Hungary

## Abstract

A S252W mutation of fibroblast growth factor receptor 2 (FGFR2), which is responsible for nearly two-thirds of Apert syndrome (AS) cases, causes retarded development of the skeleton and skull malformation resulting from premature fusion of the craniofacial sutures. We utilized a *Fgfr2^+/S252W^* mouse (a knock-in mouse model mimicking human AS) to demonstrate decreased bone mass due to reduced trabecular bone volume, reduced bone mineral density, and shortened growth plates in the long bones. *In vitro* bone mesenchymal stem cells (BMSCs) culture studies revealed that the mutant mice showed reduced BMSC proliferation, a reduction in chondrogenic differentiation, and reduced mineralization. Our results suggest that these phenomena are caused by up-regulation of p38 and Erk1/2 phosphorylation. Treatment of cultured mutant bone rudiments with SB203580 or PD98059 resulted in partial rescue of the bone growth retardation. The p38 signaling pathway especially was found to be responsible for the retarded long bone development. Our data indicate that the S252W mutation in FGFR2 directly affects endochondral ossification, resulting in growth retardation of the long bone. We also show that the p38 and Erk1/2 signaling pathways partially mediate the effects of the S252W mutation of FGFR2 on long bone development.

## Introduction

Bones are formed through one of two ossification processes, intramembranous or endochondral bone formation. Endochondral bone formation generates both cartilage and bone, and normal development of endochondral bone is dependent on the balance between chondrocyte proliferation and differentiation to achieve appositional and interstitial growth as well as vascular invasion of cartilage to allow for bone deposition.

It is widely accepted that endochondral bone formation is regulated by interdependent signaling pathways downstream of locally derived growth factors such as fibroblast growth factors (FGFs). These FGFs and their receptors are expressed in a time-dependent manner and in spatially restricted patterns in cells of the epiphyseal cartilage of developing bones. Previous studies have shown that activating mutations in the FGFRs result in disorders of endochondral bone formation characterized by a reduction in the number of proliferating chondrocytes and defective bone formation [1], [2], [3].

As one of the most severe forms of craniosynostosis, Apert syndrome (AS) is a clinically distinct condition characterized by premature fusion of coronal sutures, craniofacial anomalies, and digital syndactyly [4], [5]. Remarkably, Apert syndrome is caused by a limited number of mutations. Ninety-nine percent of reported cases have one of two missense mutations in adjacent amino acids, Ser252Trp or Pro253Arg, of fibroblast growth factor receptor 2 (FGFR2) [6]. The first mutation, S252W, is more common, occurring in 67% of patients. Both mutations affect the highly conserved linker region between the immunoglobulin-like II and III domains, resulting in increased affinity and altered specificity of fibroblast growth factor (FGF) ligand binding [7].

In addition to the abnormal intramembranous ossification that causes premature fusion of sutures, endochondral ossification is also affected in patients with Apert syndrome. Both cranial base abnormalities and epiphyseal dysplasia are observed in Apert syndrome patients [4]. The cartilage abnormalities have been reported previously in several mouse models with different mutations of FGFR2. [8], [9], [10]. Moreover, we previously detected FGFR2 expression not only in the osteoblasts [11] but also in the chondrocyte lineage either by in situ hybridization or by RT-PCR. This is consistent with other reported findings, which indicated that both intramembranous FGFR2 and endochondral bone formation are affected [12], [13]. FGFR2 can activate two primary pathways: the mitogen-activating protein kinase pathway [14], [15], [16] and the protein kinase C pathway [14], [17]. Signaling through FGFR2 regulates stem cell proliferation, affecting different lineages such as osteoblasts and chondrocytes [8], [18].

However, the effect of the activating mutation in FGFR2 on long bone development in early postnatal stages has not been systematically researched, and its mechanism is not clear. In the present study, we characterized an Apert syndrome mouse model whose genome carried a knock-in FGFR2 S252W mutation. Our results revealed that a gain-of-function mutation (S252W) in FGFR2 in mice led to decreased bone mass and proliferation of bone mesenchymal stem cells (BMSCs), as well as a reduction in BMSCs chondrogenic differentiation with reduced mineralization. Furthermore, we show that the p38 and Erk1/2 pathways may play critical roles in growth retardation of the long bones in mice with the FGFR2 S252W mutation.

## Materials and Methods

### 1. Mice

*Fgfr2^+/S252W-neo^* mice and *EIIa-Cre* mice were kindly provided by Dr. Chuxia Deng of NIDDK [19]. Both *Fgfr2^+/S252W-neo^* mice and *EIIa-Cre* mice were inbred into a C57BL/6J background by mating with C57BL/6J mice for 10 generations. *Fgfr2^+/S252W^* mice were generated by crossing male offspring of *Fgfr2^+/S252W-neo^* and female offspring of *EIIa-Cre* mice. All procedures were approved by the Institutional Animal Care and Use Committee of Daping Hospital. *Fgfr2^+/S252W^* mice and littermate controls were sacrificed by cervical dislocation for analyses.

### 2. Genotype analysis

Mice with germline transmission of FGFR2-S252W (*Fgfr2^+/S252W-neo^*) were crossed with EIIa-Cre mice to remove the neo gene in order to obtain mice with the gain-of-function mutation in FGFR2 (*Fgfr2^+/S252W^* mice), which were used for subsequent phenotype analysis. After removal of the neo gene, genotypes were determined by PCR using primer A (5′-TAG GTA GTC CAT AAC TCG G-3′) and primer B (5′-TTG ATC CAC TGG ATG TGG GGC-3′). This pair of primers amplifies a 460 bp fragment in wild-type and a 520 bp fragment in *Fgfr2^+/S252W^* mice due to the presence of a loxP site.

### 3. Radiographic imaging and micro-computed tomography

The hindlimbs of 1–4-week-old mice were harvested and stored in 4% paraformaldehyde at 4°C. High-resolution X-rays of these bones were obtained using a LoRad Selenia apparatus (Hologic, USA). The femoral cancellous bone of the distal metaphysis was scanned by micro-CT (µCT-80, Scanco Medical, Switzerland), as previously reported [3], [20]. Image acquisition was performed at 70 kV and 113 mA. Two-dimensional images were used to generate three-dimensional reconstructions and to calculate morphometric parameters defining trabecular bone mass and micro-architecture (n = 5, per samples per time point). These include BV/TV, Tb.Th, Tb.N, and Tb.Sp [21].

### 4. Tissue preparation for histology and immunohistochemistry

The tibiae were fixed in 4% paraformaldehyde overnight at 4°C, rinsed in PBS, and decalcified in 15% EDTA (pH 7.4) for 20–30 days before being embedded in paraffin as described previously (n = 3, per genotype per time point) [3]. Sections (6 µm thick) were used for H&E staining and Safranin-O/Fast green (SO/FG) staining [22].

The sections (n = 3, per genotype per time point) were deparaffinized and rinsed in PBS, followed by blocking of endogenous peroxidase activity with H_2_O_2_ at room temperature for 20 min. The sections were then washed in PBS, and nonspecific sites were blocked with 3% bovine serum albumin (BSA) in PBS at room temperature for 1 h. The sections were incubated with the primary antibodies Col2, Col10, Cbfa1, or OC (Santa Cruz Biotechnology, USA) diluted to appropriate concentrations in 2% BSA/0.1 M PBS overnight at 4°C. Sections were rinsed and exposed to a biotinylated IgG antibody for 1 hour at room temperature. After washing in PBS, slides were treated with ABC reagents (Boster, China), and signals for antibody binding were visualized with diaminobenzidine (DBA) substrate. Counterstaining was performed with methyl green.

### 5. Immunohistochemistry of proliferating-cell nuclear antigen (PCNA) and TUNEL

Immunohistochemical staining of PCNA was performed on demineralized sections using antibodies against PCNA (Epitomics, Burlingame, USA), as described previously (n = 4, per genotype per time point) [23].

TdT-mediated dUTP nick end labeling (TUNEL) was performed according to the manufacturer's instructions (Roche, Basel, Switzerland). Briefly, 6 µm sections were incubated with proteinase K working solution (Roche, Basel, Switzerland) for 30 min at 37°C. The slides were then incubated with 50 µL of the TUNEL reaction mixture at room temperature for 1 h in the dark, followed by addition of 50 µL of Converter-POD to tissues. The slides were incubated in a humidified box for 30 min at 37°C. Finally, the slides were treated with ABC reagents (Boster, China), and signals indicating antibody binding were visualized with diaminobenzidine substrate (Boster, China). Between each step, the slides were rinsed with 0.1 M PBS.

Under a light microscope, five visual fields were chosen randomly near the tibial growth plate areas. The total number of cell nuclei and the number of positively stained cells in the chosen field were then counted, and the rate of positive staining was calculated.

### 6. Bone mesenchymal stem cell culture and chondrogenic differentiation

When 6 to 8 weeks old, the mice were sacrificed by cervical dislocation. Briefly, bone marrow was flushed from the tibiae and femora under aseptic conditions using DMEM (Gibco, Invitrogen, USA) containing 10% FBS (Gibco, Invitrogen, USA) [3]. Cells were plated at a density of 5×10^5^ cells/cm^2^ and cultured in a humidified atmosphere of 5% CO_2_ at 37°C. Medium was changed initially on day 1 and every 2 days thereafter. All BMSCs assays were carried out on second passage cultures. For the proliferation assay, cells were plated at 5×10^3^ cells/well in 96-multiwell plates. Cell proliferation was detected using an *in vitro* colorimetric assay on days 2, 4, 6, 8, and 10 (n = 4, per samples per time point). BMSCs were washed with PBS and incubated in 200 µl MTT solution for 4 h at 37°C. Subsequently, MTT solution was removed, and 150 µl DMSO was added. After being mixed for 10 min at room temperature, cells were quantified spectrophotometrically at 490 nm.

For the chondrogenic differentiation assay, cells were seeded at 5×10^5^/well in 6-well plates. One day after plating, the medium was exchanged for fresh high-glucose DMEM supplemented with 10% fetal bovine serum, 10 µg/L transforming growth factor (TGF-β_1_) (Peprotech,USA), 50 µg/mL ascorbic acid, 50 mg/mL ITS (Sigma), and 10^−7^ M dexamethasone (Sigma, USA). Medium was changed every 3 days. Chondrogenic differentiation of BMSCs was assessed on days 14 and 21 using Alcian blue staining and collagen type 2 immunostaining, while mineralization was assessed using Alizarin red staining. The p38 and Erk1/2 inhibitors SB203580 and PD98059 (Selleckchem, Houston, USA) were added to the chondrogenic induction medium at a final concentration of 20 µM to compare changes in expression of osteoblast and chondrocyte marker genes.

### 7. Western blot analysis

After 14 days of culturing, BMSCs were extracted with buffer containing 50 mM Tris–HCl, pH 7.4, 150 mM NaCl, 1% Nonidet P-40, and 0.1% SDS supplemented with a cocktail of protease inhibitors (Roche, Basel, Switzerland) [24]. Protein concentrations were determined using a BCA protein assay kit (Pierce, Thermo Scientific, USA). Equal amounts of protein were fractionated by 10% SDS-PAGE and transferred to an Immobilon-P PVDF membrane (Millipore, USA). Blotted protein was probed with antibodies specific to phospho-p38 and p38, phospho-Erk1/2 and Erk1/2, phospho-AKT, and AKT (Cell Signaling Technology, USA) as described previously [11].

### 8. RT-PCR

Total RNA was prepared from BMSCs cultured for 14 and 21 days using Trizol reagent (Invitrogen, USA) according to the manufacturer's instructions. After DNase treatment, RNA was reverse-transcribed to cDNA using the ReverTra Ace® kit (TOYOBO, Osaka, Japan) according to the manufacturer's instructions. The cDNA was subjected to real-time PCR using PCR System 9700 (Applied Biosystems, Life Technologies, USA). Primer sequences and the expected sizes of the PCR products are shown in Table 1. The expression levels for each gene of interest were normalized to their corresponding β-actin values. Reactions were run in triplicate.

**Table 1:** Primers sequences used for RT–PCR in this study.

Gene	Sense primer	Antisense primer
Col2	5′-TCTTTGGCTCACTGGACTCT-3′	5′-CCCCTCCTGCTGTGAAGTTG-3′
Col10	5′-GCAGCATTACGACCCAAGAT-3′	5′- CATGATTGCACTCCCTGAAG -3′
OP	5′-TGCACCCAGATCCTATAGCC-3′	5′-TGTGGTCATGGCTTTCATTG-3′
OC	5′-TCTGACAAAGCCTTCATGTCC-3′	5′ -AAATAGTGATACCGTAGATGCG-3′

### 9. *In vitro* culture of embryonic long bones

Embryonic tibiae and the second and third phalanges were cultured according to the modified protocol described previously [11], [25], [26]. Bones were harvested from E16 mice, and four groups of cultures were set up as follows: Group 1 contained ten explants from wild-type controls, and Groups 2, 3, and 4 contained ten explants from mutants. Groups 1 and 2 were treated with 0.1% DMSO vehicle alone, group 3 was treated with 50 µM SB203580, and group 4 was treated with 50 µM PD98059. The explants were cultured in 24-well culture plates in culture medium consisting of BGJb medium (Gibco, Invitrogen, USA) containing 0.2% bovine serum albumin, 2 mM glutamine, 50 µg/mL ascorbic acid, 10 mM β-glycerophosphate, and antibiotics. The culture medium was changed every other day. The total length (TL) and the ossified tissue length (OL) were measured under a stereomicroscope (Leica, Germany) at day 0 and day 7. Changes in length of the cultured tibiae were calculated as a percentage increase (percentage increase  =  [length at day 7 - length at day 0]/length at day 0). Data are expressed as the mean ± SD, and the significance of any differences was evaluated using Student's t-test.

### 10. Statistical analysis

Statistical analyses of the data were performed using SPSS for Windows, version 13.0. Results are shown as the mean ± SD. Statistical analysis was performed using Student's t-test.

## Results

### 1. Generation of *Fgfr2^+/S252W^* mutant mice

**Figure 1:**
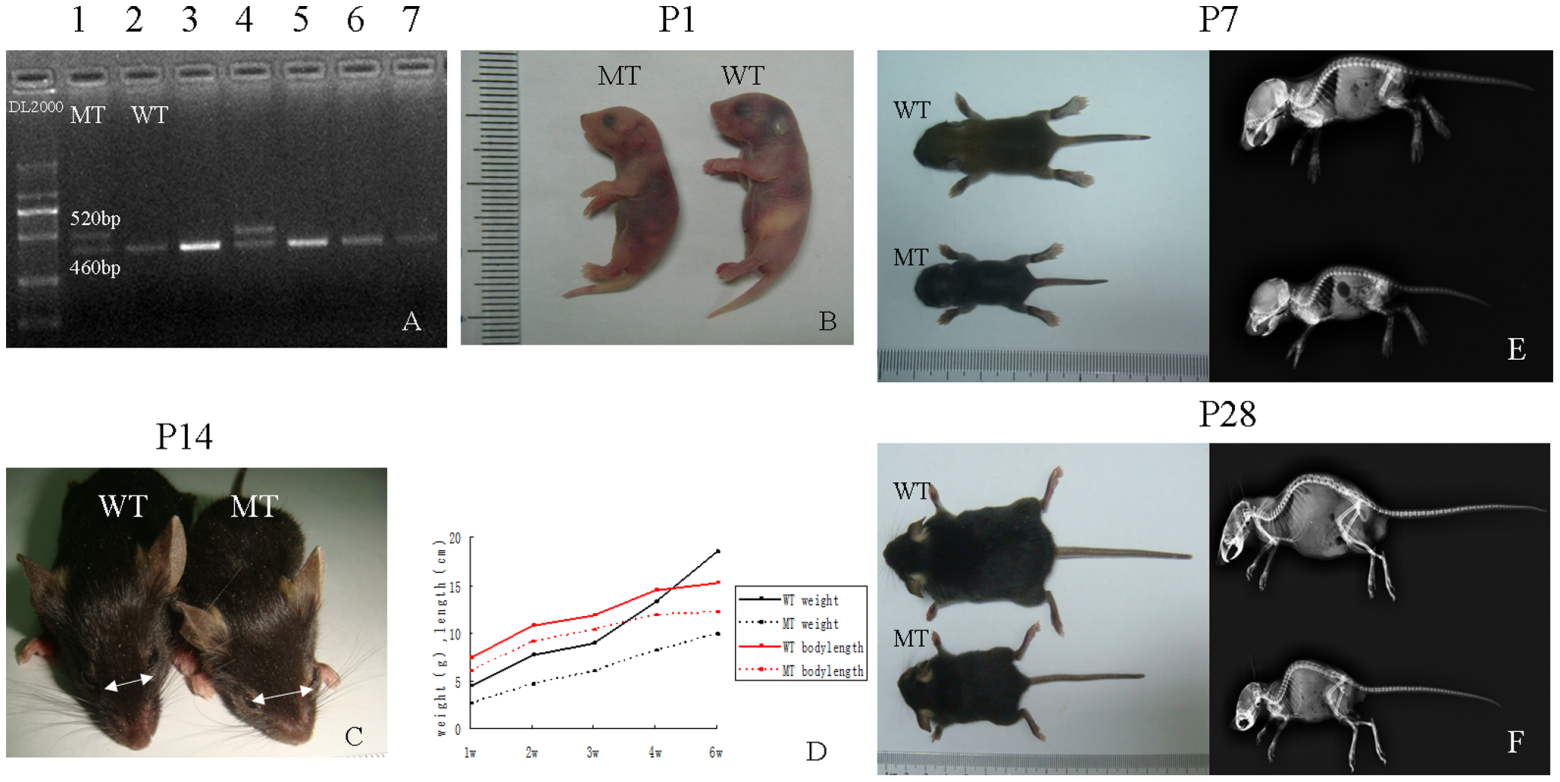
Identification and observation of bone structures in *Fgfr2^+/S252W^* mice. (A) Genotypes of a litter from *Fgfr2^+/S252W-neo^*+*EII-Cre* by PCR of tail DNA. Lanes 1 and 4 are mutant mice, showing the mutant allele after neo deletion (520 bp). Lanes 2, 3, 5, 6, and 7 are wild-type mice. (B) Gross appearance of *Fgfr2^+/S252W^* mutant (MT) and wild-type (WT) mice on postnatal day P1. (C) Top view of P14 mice. Note that the mutant mice have a smaller body size, wide-spaced eyes, and an underdeveloped midface. (D) Growth curves demonstrating significant growth retardation in the mutants. (E, F) Photographs and side view radiographs of WT and MT mice at P7 and P28. The mutant mice showed shortened anterior–posterior axes, short limbs, and overriding lower incisors.

Using a Cre-mediated knock-in method, *Fgfr2^+/S252W^* mice were generated with the S252W mutation. The *Fgfr2^+/S252W-neo^* mutant mice were crossed with *EIIa-Cre mice* [9], [11], [27] to remove the pLoxPneo gene from the germ line. After deletion of the neo cassette, PCR genotyping analysis revealed target mutant mice (Fig. 1 A).

Live *Fgfr2^+/S252W^* mice were born with the expected Mendelian frequency (25%). The mutant mice appeared abnormal at birth, being slightly smaller than wild-type littermates and exhibiting dome-shaped skulls (Fig. 1 B). The P14 mutant mice exhibited more obvious head malformations, which included widely spaced eyes and an underdeveloped midface (Fig. 1 C). However, upon gross inspection and X-ray imaging during early postnatal development (P7-P28), mutant mice exhibited significant shortening of the anterior–posterior axis accompanied by malocclusion typical of AS patients (Fig. 1 E, F). Notably, none of the mutants exhibited syndactyly as in previous studies [9], [19]. At birth, the weight of mutant mice was 90% of that of their wild-type littermates, and by three weeks of age, they were 60 to 72% smaller than controls (Fig. 1 D). Approximately 23% (9 of 39) of mutant mice died before P7, and only 41% (16 of 39) were alive after 28 days. We checked the live and naturally dead mutant mice carefully and found no significant histological abnormalities of the lung, heart, liver, or kidneys [9], [11].

### 2. The *Fgfr2^+/S252W^* mutation in mice alters bone formation

**Figure 2:**
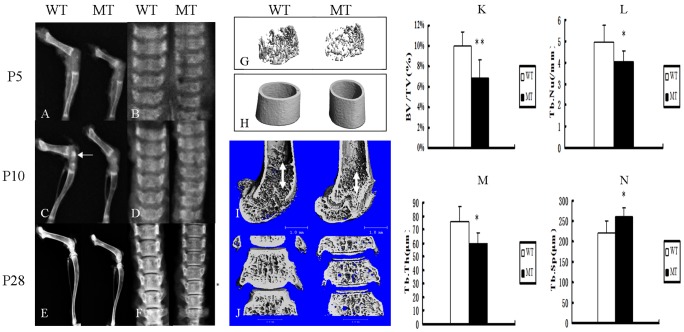
*Fgfr2^+/S252W^* mice had shortened bone length and decreased bone mass compared with wild-type mice. (A–F) X-ray analyses of femora, tibia and lumbar vertebrae from WT and MT mice at P5 (A, B), P10 (C, D) and P28 (E, F) revealed shorter bone length and lower bone density in mutant mice. At P10, the appearance of the secondary ossification centers (white arrow) was delayed in mutant tibia epiphyses. (G, H) Three-dimensional images showed reduced trabecular bone in mutant mice, thought the thickness of the diaphyses was not reduced at P14. (I, J) Micro-CT three-dimensional images of femora and lumbar vertebrae at P28 showed short and sparse trabecular. The double arrows indicate the length of trabecular. (K–N) Quantification of the structural parameters of the femoral metaphysis (P14) revealed that BV/TV, Tb.N, and Tb.Th decreased significantly, though Tb.Sp was higher in MT mice relative to WT mice. Graphs show the mean value ± SD (Student's t-test, *P<0.05, **P<0.01).

Radiological analysis of hindlimb and vertebrae from P5 to P28 mice showed that the bones of mutant mice exhibited a short length and low bone density (Fig. 2A–F). Moreover, the mutant mice showed short and sparse trabecular bones compared to wild-type mice (Fig. 2 G, I). At P10, the appearance of the secondary ossification centers was delayed in mutant tibia epiphyses, which was consistent with the results reported in human Apert patients (Fig. 2 C) [4]. Like the development of the long bone, the density of the mutant vertebrae was also obviously lower and the vertebral thickness was reduced (Fig. 2 B, D, F, J).

To determine the structural parameters of trabecular bone, we performed micro-computed tomographic (micro-CT) analysis of the bone. Trabecular bone number was reduced in both the long bones and vertebrae in mutant mice (Fig. 2 I, J), which was consistent with the result of the X-ray analysis. Conversely, there was no difference in the thickness of the bone cortex (Fig. 2 H). Quantification of the structural parameters revealed that bone volume/tissue volume (BV/TV) and trabecular number (Tb.N) were decreased by 30% and 11%, respectively. Moreover, the trabecular thickness (Tb.Th) was significantly decreased by 30% in mutant mice, though the trabecular separation (Tb.Sp) in mutant mice was significantly increased by 18% (Fig. 2K–N).

### 3. The abnormal metaphysis structure in postnatal *Fgfr2^+/S252W^* mice

Because the mutant mice were smaller than their wild type littermates and because some AS patients have short limbs [28], we observed the bone epiphyseal growth plates of mice to determine whether endochondral ossification was affected by the *Fgfr2^+/S252W^* mutation.

**Figure 3:**
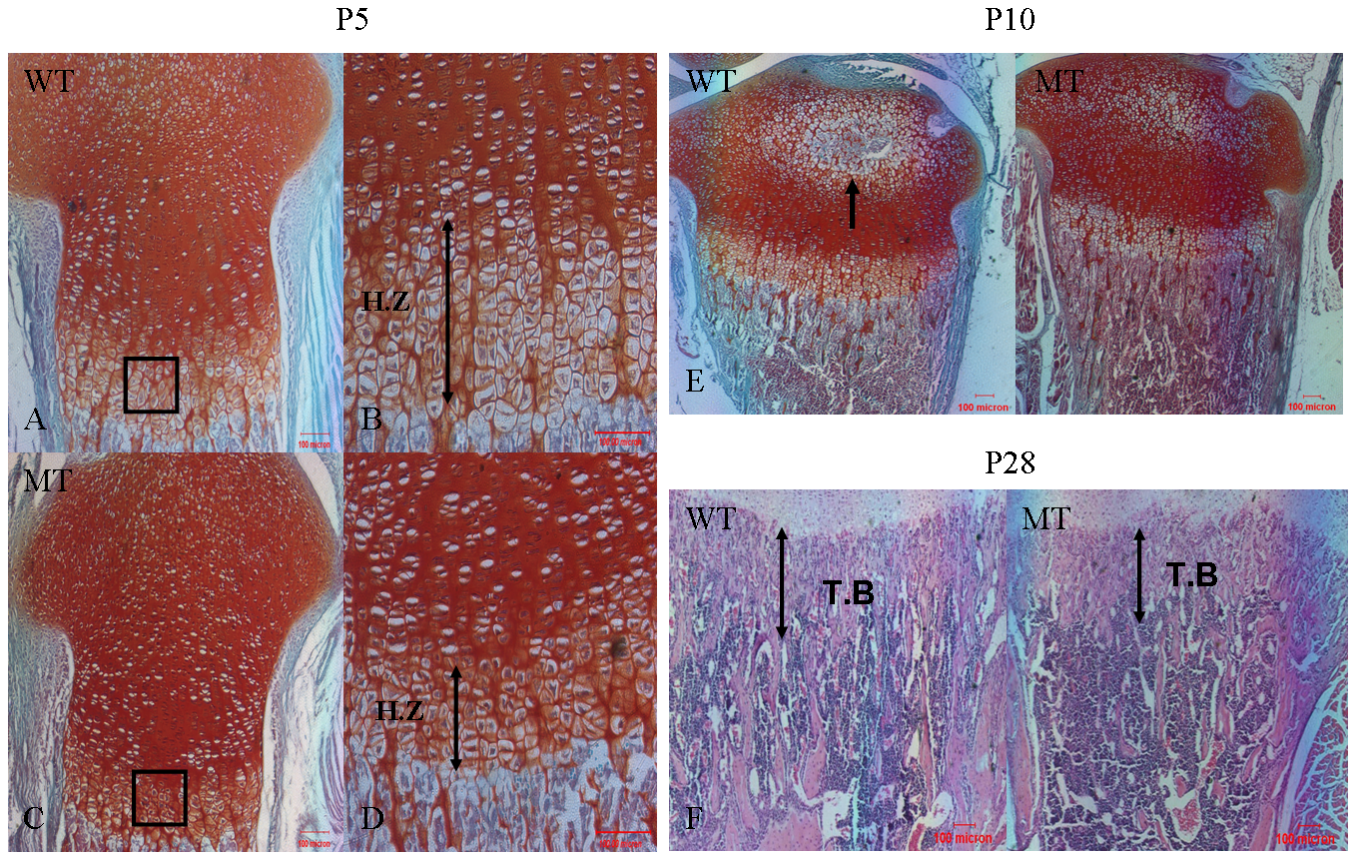
Histochemical analysis of tibia from wild-type mice and *Fgfr2^+/S252W^* mice. (A, C) Safranin-O/Fast green (SO/FG)-stained sections of the proximal tibia of P5 mice showing the epiphyseal growth plate. The height of the growth plate was shorter in mutant mice. (B, D) Enlarged view of the boxed regions in A and C showing the morphology of the hypertrophic zone. The length of the hypertrophic zone was shorter, indicated by double arrows. Notably, the volume of hypertrophic chondrocytes was smaller, with disorganized arrangement. (E) SO/FG staining shows the appearance of the secondary ossification center (arrow) in the tibia of WT mice but not in MT mice at P10. (F) H&E staining shows that the length of the trabecular bone (double arrows) in the tibia was shorter in MT mice. (A, C) 100×; (B, D) 200×; (E, F) 40×. H.Z, hypertrophic zone; T.B, trabecular bone. Scale bars  = 100 µm.

Our analysis revealed that at the early postnatal stage P5, the heights of the growth plates correlated with the smaller size of the mutants, and marked disorganization of the growth plates with subtle irregularity of the hypertrophic zone was found (Fig. 3A–D). Fig. 3 E shows that at P10, secondary ossification centers appear in the tibial epiphyses of wild-type mice but not in the mutants, which was consistent with the X-ray examination. The bones of *Fgfr2^+/S252W^* mice also contained significantly less trabecular bone and in some instances were nearly devoid of trabecular bone. Histological analysis showed that trabecular zone length and width were reduced, resulting in a decrease in metaphyseal area (Fig. 3 F). This observation suggests that the reduced size of the growth plates in mutant mice is most likely due to retarded endochondral ossification.

### 4. The effects of enhanced FGFR2 function on proliferation and differentiation at the growth plate

**Figure 4:**
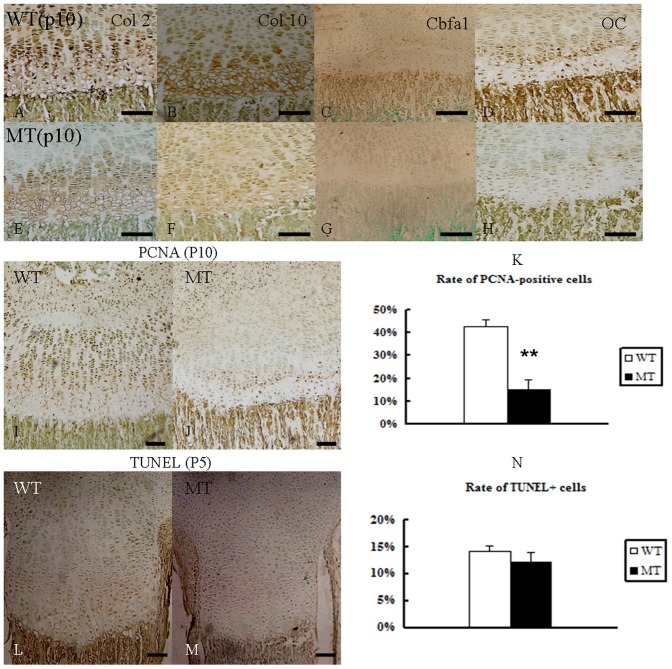
Abnormal proliferation and differentiation at the growth plate in *Fgfr2^+/S252W^* mice from P10. (A–H) Col 2, Col 10, Cbfa1 and OC immunoreactivity in the tibia epiphyses. Immunolocalization (brown color) of both chondrocyte and osteocyte markers in the entire epiphyseal area were reduced in MT mice at P10 (WT: A–D, MT: E–H). (I, J) PCNA immunohistochemistry revealed a significantly reduced number of proliferating chondrocytes in the tibia growth plate of P10 MT mice. (K) The percentage of PCNA-labeled cells was reduced in the growth plates of the MT mice. (L, M) TUNEL assay revealed no difference in apoptosis in the tibia growth plate of the two types at P5. (N) The Percentage of TUNEL+ labeled cells was similar in the two groups. Data are presented as the mean ±SD. **P<0.01. Scale bars  = 100 µm.

FGFR2 expression in the epiphyses of long bones was identified in earlier studies [11], [12]. We therefore examined the effect of the FGFR2 S252W mutation on Col 2, Col 10, Cbfa1, and OC expression in the postnatal (P10) tibial growth plate. The expression of both Col 2 and Col 10 in chondrocytes was reduced in area and intensity, reflecting the decreased length of this zone (Fig. 4 A, B, E, F). The expression of the osteocyte markers Cbfa1 and OC were correspondingly reduced in the mutant (Fig. 4 C, D, G, H). The obvious decrease in expression of these proteins in mutant mice prompted us to speculate that gain of function of FGFR2 retards metaphyseal chondrogenic and osteogenic differentiation. The number of PCNA-positive proliferating chondrocytes in the growth plates of the proximal tibia was significantly decreased in *Fgfr2^+/S252W^* mice (Fig. 4 I, J), indicating that chondrocyte proliferation was decreased in *Fgfr2^+/S252W^* mice. To determine whether the *Fgfr2^+/S252W^* mutation affects apoptosis, we examined *Fgfr2^+/S252W^* mutant and control mice using the TUNEL assay. Our examination of wild-type and mutant mice at P5 revealed similar levels of apoptosis in the tibial growth plate (Fig. 4 K, L), suggesting that apoptosis may not be a major factor at this stage of long bone development.

### 5. Gain-of-function mutation of FGFR2 has different effects on the proliferation and chondrogenic differentiation of BMSCs

Mesenchymal stem cells (MSCs) have the potential to differentiate into different lineages, including osteoblasts, chondrocytes, and adipocytes [29], [30], [31]. Because we have shown that *Fgfr2^+/S252W^* mice exhibited retarded endochondral ossification, we further explored the mechanism underlying the altered bone formation in *Fgfr2^+/S252W^* mice by culturing BMSCs under **c**hondrogenic differentiation inducing conditions over the long term, evaluating changes in cell proliferation and differentiation.

**Figure 5:**
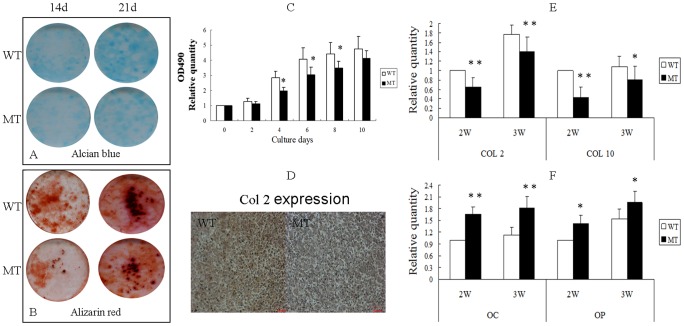
Effects of activated FGFR2 on the proliferation and differentiation of BMSCs. (A) Alcian blue staining showed reduced numbers of blue-staining cells in cultured BMSCs from MT mice compared with WT on days 14 and 21. (B) Alizarin red staining of the mineralized osteoblasts showed reduced numbers of mineralized nodules on days 14 and 21 in MT mice. (C) MTT proliferation assays showed reduced proliferation of BMSCs from MT mice. (D) Histochemical staining shows that Col 2 expression was lower in MT mice than in WT after chondrogenic induction. (E) Relative expression of early chondrogenic differentiation genes measured by RT-PCR. The expression levels of *Col2* and *Col10* mRNA in differentiated BMSCs were markedly reduced in BMSCs from MT mice at 2 and 3 weeks. (F) Relative expression of late chondrogenic differentiation genes measured by RT-PCR. The expression levels of *OC* and *OP* mRNA in differentiated BMSCs were increased cells from MT mice at 2 and 3 weeks. Graphs show mean ±SD (Student's t-test, *P<0.05, **P<0.01).

We found that cell proliferation was reduced in BMSCs derived from *Fgfr2^+/S252W^* mice compared with wild-type BMSCs (Fig. 5 C). To examine the differentiation potential of BMSCs, we compared Alcian blue and Alizarin red staining in BMSCs isolated from both wild-type and *Fgfr2^+/S252W^* mice cultured in chondrogenic differentiation medium. After 14 and 21 days of culture, the BMSCs from *Fgfr2^+/S252W^* mice showed reduced numbers of blue-staining cells (Fig. 5 A), showing that the chondrogenic activity of BMSCs was significantly reduced in *Fgfr2^+/S252W^* mice. Consistent with the above results, FGFR2 had the same effect on mutated BMSCs as revealed by Col2 histochemical staining, suggesting that the gain-of-function mutation in FGFR2 inhibits the early chondrogenic differentiation of BMSCs under these experimental conditions (Fig. 5 D). Moreover, *Fgfr2^+/S252W^* BMSCs developed fewer mineralized colonies and exhibited reduced alizarin red staining (Fig. 5 B). These results suggest that the gain-of-function mutation of FGFR2 also blocked mineralization.

RNA harvested from BMSCs after 21 days of culture was used to measure the expression of genes related to chondrogenic differentiation by real-time PCR. As shown in Figure 5 E and F, the early stage of chondrocyte expression levels of *Col2* and *Col10* in BMSCs were all reduced in comparison with wild-type mice. However, the expression levels of *OC* and *OP*, which are markers of the late stage of chondrocyte [32], were increased. These results further indicate that gain-of-function mutation of FGFR2 could influence different stages of chondrogenic differentiation of BMSCs.

### 6. The gain-of-function mutation in FGFR2 affects proliferation and differentiation of BMSCs through the Erk1/2 and P38 pathways

To investigate the molecular basis or signaling pathways involved in the abnormal proliferation and differentiation observed in mutant BMSCs, we used western blotting to check the phosphorylated and total protein levels of Erk1/2, p38 and AKT [9], [14], [33]. These levels were normalized to β-actin levels.

**Figure 6:**
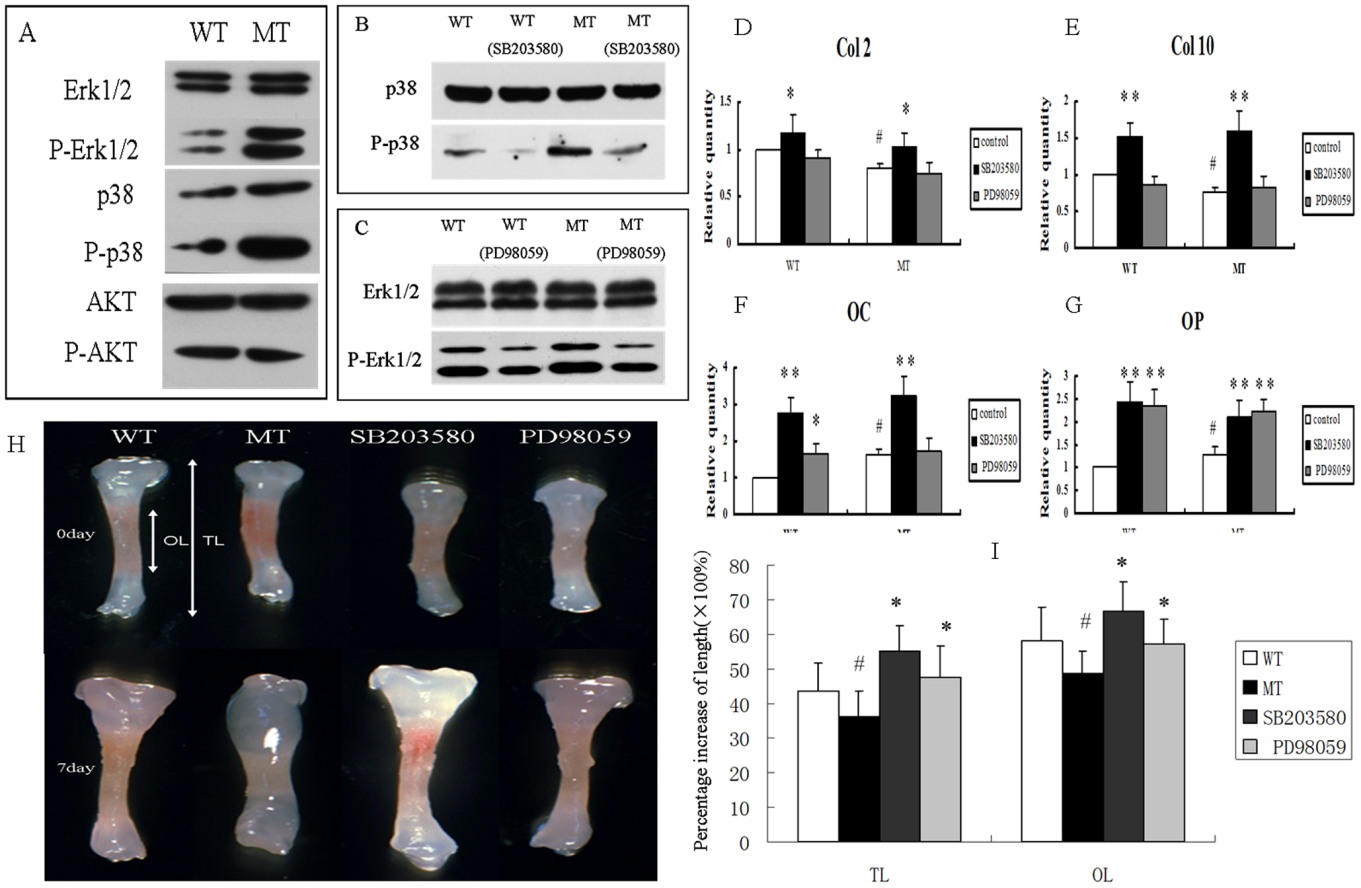
p38 and Erk1/2 pathway participated in the regulation of BMSCs by FGFR2. (A) Western blotting demonstrated that levels of phospho-Erk1/2 and phospho-p38 were both increased in cultured BMSCs from MT mice. There was, however, no change in phospho-AKT. (B, C) The p38 and Erk1/2 pathways were inhibited by SB203580 and PD98059, respectively. (D–G) Relative expression of genes in BMSCs treated with SB203580 or PD98059 for 21 days. The expression levels of *Col2*, *Col10*, *OC*, and *OP* were significantly increased in both WT and MT BMSCs treated with SB203580; however, only *OC* and *OP* were increased after PD98059 treatment. (Student's t-test, *P<0.05, **P<0.01 versus untreated BMSCs, ^#^ *P*<0.05 versus wild-type BMSCs.). (H) Inhibition of p38 and Erk1/2 pathway by SB203580 and PD98059 rescued the growth retardation in cultured mutant tibia bones. The white double-headed arrows represent the total length (TL) and ossified tissue length (OL). (I) Percentage increases in TL and OL of phalange bones after culture for 7 days. (Student's t-test, ^#^P<0.05 versus wild-type *P<0.05 versus mutant type.)

The results showed increased phosphorylation of Erk1/2 and p38 in *Fgfr2^+/S252W^* mice BMSCs, which indicated that Erk1/2 and p38 signaling may participate in the regulation of endochondral bone formation by FGFR2. However, phosphorylated AKT and AKT protein levels did not show any obvious increase in mutant mice (Fig. 6 A).

To determine whether Erk1/2 and p38 were indeed involved in the phenotypes of *Fgfr2^+/S252W^* mice, we investigated the effects of Erk1/2 and p38 in cultured BMSCs using specific inhibitors against either Erk1/2 or p38 (Fig. 6 B, C). BMSCs were treated with the p38 inhibitor SB203580 or the Erk1/2 inhibitor PD98059 for 21 days in chondrogenic differentiation inducing medium. The expression of marker genes was again measured using real-time PCR. As shown in Fig. 6D–G, the levels of *Col2*, *Col10*, *OC*, and *OP* mRNAs were significantly up-regulated in BMSCs from both wild-type and *Fgfr2^+/S252W^* mice by SB203580 (21 days). Treatment with PD98059 (21 days) increased the levels of the terminal chondrogenic differentiation genes *OC* and *OP* in both types but had little effect on the levels of the early chondrogenic differentiation marker genes *Col2* and *Col10*.

### 7. Retardation of bone growth caused by gain-of-function mutation of FGFR2 is rescued by treatment with SB203580 or PD98059

We then investigated whether p38 and Erk1/2 are involved in the abnormal development of long bones observed *in vitro*. The mutant bones were found to be shorter than the bones from wild-type mice at the beginning of the study. After seven days in culture, compared to the wild-type bones, the mutant long bones showed significant growth inhibition (Fig. 6H). However, the growth retardation observed in both total length and ossified tissue length in cultured mutant embryonic tibiae were significantly rescued by inhibitors of p38 and Erk1/2. We further measured the percentage increases of phalange in TL and OL after culture for 7 days (Fig. 6I), concluding that TL and OL length were more increased by the inhibitor SB203580.

## Discussion

Apert syndrome (AS) is one of the most severe forms of craniosynostosis(CS) and is mainly characterized by premature fusion of the coronal suture and severe syndactyly of the hands or feet. Activation of osteoblastic bone anabolism in the calvarial sutures is considered to be the essential pathologic condition underlying mutant FGFR2-related craniofacial dysostosis [9], [17], [19], [34]. To systematically examine the significance of endochondral ossification, we experimented with a mouse model (*Fgfr2^+/S252W^*) mimicking human AS.

As previous described [19], our mice were likely to exhibit effects of the mutant FGFR2 on endochondral ossification, showing skull malformations and shortening of the anterior–posterior axis similar to bone defects seen in patients with Apert syndrome [9], [10], [11], [34]. By analyzing our *Fgfr2^+/S252W^* mouse model, we found that the FGFR2 S252W mutation led to reduced endochondral ossification. To exclude the potential effects of malnutrition caused by malocclusion on bone development, protruding incisors of mutant mice were cut, but the mutant mice still exhibited shorter stature.

We further explored the structure of the long bone by X-ray and Micro-CT, finding that activated FGFR2 led to reduced bone mass and weaker structure. Both bone volume (BV) and bone mineral density (BMD) were reduced in the femora of *Fgfr2^+/S252W^* mice. Structural analysis of bone by micro-CT also demonstrated a marked reduction in trabecular number in the metaphysis of the femora and vertebrae from mutant mice at P28. However, unlike *Fgfr2^cko^* mice(Fgfr2 fox/Δ,Dermo1Cre) [28], which showed a dramatic reduction in the thickness of the femoral bone cortex, *Fgfr2^+/S252W^* mice was the same as the wild-type.

The growth plate not only is an important area to achieve longitudinal growth of the long bone but also regulates the longitudinal bone growth rate during endochondral bone development [35]. Chen [19] concluded that in some older mice (P10 and older), the mutant growth plates had slightly shorter columns of proliferating chondrocytes, but further mechanisms of retarded endochondral ossification were not determined. There are some controversies regarding the chondrocyte differentiation in the cranial base. Yin [11] found a shortened cranial base and revealed decreased *Col10* expression in the cranial base in P1 *Fgfr2^+/P253R^* mutant mice, suggesting decreased chondrocyte differentiation. However, Wang [9] and Nagata [10] found up-regulation of chondrocyte-related gene expression and reduced chondrocytic maturation and hypertrophy in the cranial base. Moreover, detailed histopathological study of the effects of the *FGFR2 S252W* mutation in long bone has not been previously conducted.

Our *Fgfr2^+/S252W^* mice showed shorter growth plate in early postnatal stages. The marked disorganization of the hypertrophic zone was observed at P5, and the irregularity and small size of the hypertrophic chondrocyte was consistent with defects found in other *Fgfr2^+/S252W^* [9], [11] or *Fgfr2^-/-^* [8], [28] mouse models. Our findings that the expression of Col2, Col10, Cbfa1, and OC were immediately down-regulated reflected the reduced chondrocyte proliferation, chondrogenic and osteogenic differentiation in the shortened metaphysis. These expression levels differ from the initial levels in the cranial base. Furthermore, this abnormal histological structure led to the delayed appearance of secondary ossification centers and delayed bone growth. However, mice carrying *FGFR2* mutations showed no obvious difference in metaphysis chondrocyte apoptosis, which was opposite to the observed increases in cranial suture osteoblast apoptosis made by Chen [19] and Mansukhani [36].

Our results showed that the precise regulation in different organs or cells by FGFR2 mutation was complex and unique. The FGFR2 mutation in cranial suture **c**hondrocytes, which increased cartilage proliferation and differentiation[9], [10], ultimately caused craniosynostosis. However, our data suggested that FGFR2 mutation in the metaphysis caused developmental disorders of long bone, resulting in shorter length and reduced bone mass.

During endochondral ossification, mesenchymal cells first differentiate into chondrocytes to form a cartilaginous anlage, which is eventually replaced by bone tissue. To further explore the mechanism underlying the altered bone formation in *Fgfr2^+/S252W^* mice, we cultured BMSCs to evaluate changes in cell proliferation and differentiation. Although FGF signaling has been found to promote cell proliferation in bone marrow-derived MSCs [37], the specific function of the FGFR2 S252W mutation in this process was not identified. Interestingly, the proliferation of BMSCs of Fgfr2+/S252W mice was decreased in the early days of culturing (P4–P8). There has been controversy regarding the proliferation and differentiation of BMSCs resulting from gain-of-function mutations in FGFR2. Miraoui [14] showed that WT and MT FGFR2 increased C3H10T1/2 cell proliferation but not survival. Both WT and MT FGFR2 increased early and late osteoblast gene expression and matrix mineralization. In both cell types, MT FGFR2 was more effective than WT FGFR2. Genetic manipulation of murine osteoblasts indicated that gain-of-function mutations of FGFR2 can either reduce [36], [38] or increase osteoblast differentiation [9], [11], [19]. Unlike in previous osteoblast induction conditions, we used *in vitro* chondrocyte induction conditions to imitate BMSCs endochondral ossification. In both wild-type and mutant BMSCs, we measured the expression of *Col2* [9], and combined with the results of Alcian blue staining, Alizarin red staining and RT-PCR, we conclude that gain-of-function mutations of FGFR2 can both weaken BMSCs chondrogenic differentiation and block their terminal mineralization [3], [36]. We also found that the expression of early chondrogenic differentiation genes in mutant mice was reduced, while the expression of terminal chondrogenic differentiation genes was higher. Under chondrocyte induction condition, the S252W mutation of FGFR2 was verified to delay early stages of chondrogenic differentiation and promote terminal chondrogenic differentiation while blocking matrix mineralization. Immunohistochemistry revealed the down-regulated expression of *Cbfa1*, *OC* in mutant metaphysis, FGFR2 was important for different stages of osteoblast maturation. Our examination of osteogenic markers showed that activation of FGFR2 in BMSCs accelerated terminal chondrogenic differentiation [14], [32] but that activation of FGFR2 in *metaphysis primary bone trabecular* osteoblasts delayed osteogenic differentiation [39].

A key aim was to identify the signaling mechanisms that mediate the positive effect of activated FGFR2 on bone development in mesenchymal stem cells, which were not clear. Recent studies suggested that MAPK, PKC, and AKT signaling pathways may play an important role in osteogenic and chondrogenic differentiation [11], [16], [33], [40], [41], [42], [43]. In agreement with results published by Wang [16], we speculated that the p38 or Erk1/2 signaling pathway may be involved in the pathogenesis of the skeletal phenotypes resulting from FGFR2 mutation.

We compared the changes in gene expression when the p38 or Erk1/2 signaling pathway was inhibited by SB203580 or PD98059 in BMSCs. Under chondrocyte induction conditions, the already reduced gene expression in *Fgfr2^+/S252W^* mice was recovered after inhibition of p38 signaling. However, there were no obvious changes in *Col 2* and *Col 10* under treatment with the Erk1/2 pathway inhibitor. All these results suggested that p38 and Erk1/2 have distinct roles in the process of chondrogenic differentiation. The p38 signaling pathway influences the whole endochondral ossification processes [44], [45], while the Erk1/2 promotes the late stage of chondrogenic differentiation of BMSCs. MAPK acts as a negative regulator of chondrogenesis, and the FGF-induced growth arrest of chondrocytes is partially mediated by activation of the p38 and Erk1/2 pathway [3], [14], [16], [33]. To provide direct evidence for the involvement of p38 and Erk1/2 in the pathogenesis of long bone development resulting from the S252W mutation in FGFR2, we used *in vitro* bone culture to examine the effects of SB203580 and PD98059 on the development of bones from Fgfr2^+/S252W^ mice. Our data revealed that inhibition of p38 activity significantly rescued bone growth retardation. Meanwhile, inhibition of the Erk1/2 pathway also partially restored the retarded endochondral ossification of cultured mutant bone [46]. We therefore speculate that these pathways may also be involved in the pathogenesis of skeletal phenotypes resulting from the FGFR2 mutation, and during endochondral ossification in AS, p38 appears to be more important than Erk1/2, affecting the whole process of chondrogenic differentiation.

In summary, to investigate the role of activated FGFR2 on endochondral bone formation, we utilized a mouse model mimicking human Apert syndrome resulting from the FGFR2 S252W mutation. We systematically compared the *Fgfr2^+/S252W^* mutants with wild-type littermates to provide new insight into the regulation of fetal bone development. The present data identified FGFR2 as a regulatory molecule that inhibits BMSC chondrogenic proliferation, differentiation, and mineralization. Moreover, we provide evidence that the p38 and Erk1/2 pathways are involved in the pathogenesis of skeletal abnormalities induced by the FGFR2 S252W mutation. The p38 signaling pathway in particular influences the whole process of chondrogenic differentiation, and its inhibitor can rescue growth retardation of Fgfr2+/S252W mutants. These results reveal that activated FGFR2 plays an important role in murine endochondral ossification and provide insights into the molecular signals involved in the process of endochondral ossification of murine BMSCs induced by FGFR2 activation.

## References

[1] Li C, Chen L, Iwata T, Kitagawa M, Fu XY, et al (1999) A Lys644Glu substitution in fibroblast growth factor receptor 3 (FGFR3) causes dwarfism in mice by activation of STATs and ink4 cell cycle inhibitors. Hum Mol Genet 8: 35–44. 9887329 10.1093/hmg/8.1.35

[2] Muenke M, Schell U (1995) Fibroblast-growth-factor receptor mutations in human skeletal disorders. Trends Genet 11: 308–313. 8585128 10.1016/s0168-9525(00)89088-5

[3] Su N, Sun Q, Li C, Lu X, Qi H, et al (2010) Gain-of-function mutation in FGFR3 in mice leads to decreased bone mass by affecting both osteoblastogenesis and osteoclastogenesis. Hum Mol Genet 19: 1199–1210. 20053668 10.1093/hmg/ddp590 PMC3115638

[4] Cohen MM Jr, Kreiborg S (1993) Skeletal abnormalities in the Apert syndrome. Am J Med Genet 47: 624–632. 8266987 10.1002/ajmg.1320470509

[5] Cohen MM Jr (1988) Craniosynostosis update 1987. Am J Med Genet Suppl 4: 99–148. 10.1002/ajmg.1320310514 3144990

[6] Park WJ, Theda C, Maestri NE, Meyers GA, Fryburg JS, et al (1995) Analysis of phenotypic features and FGFR2 mutations in Apert syndrome. Am J Hum Genet 57: 321–328. 7668257 PMC1801532

[7] Yu K, Ornitz DM (2001) Uncoupling fibroblast growth factor receptor 2 ligand binding specificity leads to Apert syndrome-like phenotypes. Proc Natl Acad Sci U S A 98: 3641–3643. 11274381 10.1073/pnas.081082498 PMC33332

[8] Eswarakumar VP, Monsonego-Ornan E, Pines M, Antonopoulou I, Morriss-Kay GM, et al (2002) The IIIc alternative of Fgfr2 is a positive regulator of bone formation. Development 129: 3783–3793. 12135917 10.1242/dev.129.16.3783

[9] Wang Y, Xiao R, Yang F, Karim BO, Iacovelli AJ, et al (2005) Abnormalities in cartilage and bone development in the Apert syndrome FGFR2(+/S252W) mouse. Development 132: 3537–3548. 15975938 10.1242/dev.01914

[10] Nagata M, Nuckolls GH, Wang X, Shum L, Seki Y, et al (2011) The primary site of the acrocephalic feature in Apert Syndrome is a dwarf cranial base with accelerated chondrocytic differentiation due to aberrant activation of the FGFR2 signaling. Bone 48: 847–856. 21129456 10.1016/j.bone.2010.11.014

[11] Yin L, Du X, Li C, Xu X, Chen Z, et al (2008) A Pro253Arg mutation in fibroblast growth factor receptor 2 (Fgfr2) causes skeleton malformation mimicking human Apert syndrome by affecting both chondrogenesis and osteogenesis. Bone 42: 631–643. 18242159 10.1016/j.bone.2007.11.019

[12] Lazarus JE, Hegde A, Andrade AC, Nilsson O, Baron J (2007) Fibroblast growth factor expression in the postnatal growth plate. Bone 40: 577–586. 17169623 10.1016/j.bone.2006.10.013

[13] Rice DP, Rice R, Thesleff I (2003) Fgfr mRNA isoforms in craniofacial bone development. Bone 33: 14–27. 12919696 10.1016/s8756-3282(03)00163-7

[14] Miraoui H, Oudina K, Petite H, Tanimoto Y, Moriyama K, et al (2009) Fibroblast growth factor receptor 2 promotes osteogenic differentiation in mesenchymal cells via ERK1/2 and protein kinase C signaling. J Biol Chem 284: 4897–4904. 19117954 10.1074/jbc.M805432200

[15] Ornitz DM, Itoh N (2001) Fibroblast growth factors. Genome Biol 2: REVIEWS3005. 11276432 10.1186/gb-2001-2-3-reviews3005 PMC138918

[16] Wang Y, Sun M, Uhlhorn VL, Zhou X, Peter I, et al (2010) Activation of p38 MAPK pathway in the skull abnormalities of Apert syndrome Fgfr2(+P253R) mice. BMC Dev Biol 10: 22. 20175913 10.1186/1471-213X-10-22 PMC2838826

[17] Lemonnier J, Delannoy P, Hott M, Lomri A, Modrowski D, et al (2000) The Ser252Trp fibroblast growth factor receptor-2 (FGFR-2) mutation induces PKC-independent downregulation of FGFR-2 associated with premature calvaria osteoblast differentiation. Exp Cell Res 256: 158–167. 10739663 10.1006/excr.2000.4820

[18] Ornitz DM, Marie PJ (2002) FGF signaling pathways in endochondral and intramembranous bone development and human genetic disease. Genes Dev 16: 1446–1465. 12080084 10.1101/gad.990702

[19] Chen L, Li D, Li C, Engel A, Deng CX (2003) A Ser252Trp [corrected] substitution in mouse fibroblast growth factor receptor 2 (Fgfr2) results in craniosynostosis. Bone 33: 169–178. 14499350 10.1016/s8756-3282(03)00222-9

[20] Montero A, Okada Y, Tomita M, Ito M, Tsurukami H, et al (2000) Disruption of the fibroblast growth factor-2 gene results in decreased bone mass and bone formation. J Clin Invest 105: 1085–1093. 10772653 10.1172/JCI8641 PMC300831

[21] Hurley MM, Okada Y, Xiao L, Tanaka Y, Ito M, et al (2006) Impaired bone anabolic response to parathyroid hormone in Fgf2-/- and Fgf2+/− mice. Biochem Biophys Res Commun 341: 989–994. 16455048 10.1016/j.bbrc.2006.01.044

[22] Su N, Yang J, Xie Y, Du X, Lu X, et al (2008) Gain-of-function mutation of FGFR3 results in impaired fracture healing due to inhibition of chondrocyte differentiation. Biochem Biophys Res Commun 376: 454–459. 18789890 10.1016/j.bbrc.2008.08.165

[23] Iwaki A, Jingushi S, Oda Y, Izumi T, Shida JI, et al (1997) Localization and quantification of proliferating cells during rat fracture repair: detection of proliferating cell nuclear antigen by immunohistochemistry. J Bone Miner Res 12: 96–102. 9240731 10.1359/jbmr.1997.12.1.96

[24] Rousseau F, Bonaventure J, Legeai-Mallet L, Pelet A, Rozet JM, et al (1994) Mutations in the gene encoding fibroblast growth factor receptor-3 in achondroplasia. Nature 371: 252–254. 8078586 10.1038/371252a0

[25] Jin M, Yu Y, Qi H, Xie Y, Su N, et al (2012) A novel FGFR3-binding peptide inhibits FGFR3 signaling and reverses the lethal phenotype of mice mimicking human thanatophoric dysplasia. Hum Mol Genet 21: 5443–5455. 23014564 10.1093/hmg/dds390 PMC3657479

[26] Chen L, Li C, Qiao W, Xu X, Deng C (2001) A Ser(365)–>Cys mutation of fibroblast growth factor receptor 3 in mouse downregulates Ihh/PTHrP signals and causes severe achondroplasia. Hum Mol Genet 10: 457–465. 11181569 10.1093/hmg/10.5.457

[27] Lakso M, Pichel JG, Gorman JR, Sauer B, Okamoto Y, et al (1996) Efficient in vivo manipulation of mouse genomic sequences at the zygote stage. Proc Natl Acad Sci U S A 93: 5860–5865. 8650183 10.1073/pnas.93.12.5860 PMC39152

[28] Yu K, Xu J, Liu Z, Sosic D, Shao J, et al (2003) Conditional inactivation of FGF receptor 2 reveals an essential role for FGF signaling in the regulation of osteoblast function and bone growth. Development 130: 3063–3074. 12756187 10.1242/dev.00491

[29] Bianco P, Riminucci M, Gronthos S, Robey PG (2001) Bone marrow stromal stem cells: nature, biology, and potential applications. Stem Cells 19: 180–192. 11359943 10.1634/stemcells.19-3-180

[30] Marie PJ, Fromigue O (2006) Osteogenic differentiation of human marrow-derived mesenchymal stem cells. Regen Med 1: 539–548. 17465848 10.2217/17460751.1.4.539

[31] Phinney DG, Prockop DJ (2007) Concise review: mesenchymal stem/multipotent stromal cells: the state of transdifferentiation and modes of tissue repair–current views. Stem Cells 25: 2896–2902. 17901396 10.1634/stemcells.2007-0637

[32] Enomoto H, Enomoto-Iwamoto M, Iwamoto M, Nomura S, Himeno M, et al (2000) Cbfa1 is a positive regulatory factor in chondrocyte maturation. J Biol Chem 275: 8695–8702. 10722711 10.1074/jbc.275.12.8695

[33] Xiao G, Jiang D, Gopalakrishnan R, Franceschi RT (2002) Fibroblast growth factor 2 induction of the osteocalcin gene requires MAPK activity and phosphorylation of the osteoblast transcription factor, Cbfa1/Runx2. J Biol Chem 277: 36181–36187. 12110689 10.1074/jbc.M206057200

[34] Eswarakumar VP, Horowitz MC, Locklin R, Morriss-Kay GM, Lonai P (2004) A gain-of-function mutation of Fgfr2c demonstrates the roles of this receptor variant in osteogenesis. Proc Natl Acad Sci U S A 101: 12555–12560. 15316116 10.1073/pnas.0405031101 PMC515096

[35] Olsen BR, Reginato AM, Wang W (2000) Bone development. Annu Rev Cell Dev Biol 16: 191–220. 11031235 10.1146/annurev.cellbio.16.1.191

[36] Mansukhani A, Bellosta P, Sahni M, Basilico C (2000) Signaling by fibroblast growth factors (FGF) and fibroblast growth factor receptor 2 (FGFR2)-activating mutations blocks mineralization and induces apoptosis in osteoblasts. J Cell Biol 149: 1297–1308. 10851026 10.1083/jcb.149.6.1297 PMC2175120

[37] Martin I, Muraglia A, Campanile G, Cancedda R, Quarto R (1997) Fibroblast growth factor-2 supports ex vivo expansion and maintenance of osteogenic precursors from human bone marrow. Endocrinology 138: 4456–4462. 9322963 10.1210/endo.138.10.5425

[38] Mansukhani A, Ambrosetti D, Holmes G, Cornivelli L, Basilico C (2005) Sox2 induction by FGF and FGFR2 activating mutations inhibits Wnt signaling and osteoblast differentiation. J Cell Biol 168: 1065–1076. 15781477 10.1083/jcb.200409182 PMC2171836

[39] Jacob AL, Smith C, Partanen J, Ornitz DM (2006) Fibroblast growth factor receptor 1 signaling in the osteo-chondrogenic cell lineage regulates sequential steps of osteoblast maturation. Dev Biol 296: 315–328. 16815385 10.1016/j.ydbio.2006.05.031 PMC2077084

[40] Lemonnier J, Hay E, Delannoy P, Fromigue O, Lomri A, et al (2001) Increased osteoblast apoptosis in apert craniosynostosis: role of protein kinase C and interleukin-1. Am J Pathol 158: 1833–1842. 11337381 10.1016/S0002-9440(10)64139-9 PMC1891948

[41] Lemonnier J, Hay E, Delannoy P, Lomri A, Modrowski D, et al (2001) Role of N-cadherin and protein kinase C in osteoblast gene activation induced by the S252W fibroblast growth factor receptor 2 mutation in Apert craniosynostosis. J Bone Miner Res 16: 832–845. 11341328 10.1359/jbmr.2001.16.5.832

[42] Shukla V, Coumoul X, Wang RH, Kim HS, Deng CX (2007) RNA interference and inhibition of MEK-ERK signaling prevent abnormal skeletal phenotypes in a mouse model of craniosynostosis. Nat Genet 39: 1145–1150. 17694057 10.1038/ng2096

[43] Su N, Du X, Chen L (2008) FGF signaling: its role in bone development and human skeleton diseases. Front Biosci 13: 2842–2865. 17981758 10.2741/2890

[44] Raucci A, Laplantine E, Mansukhani A, Basilico C (2004) Activation of the ERK1/2 and p38 mitogen-activated protein kinase pathways mediates fibroblast growth factor-induced growth arrest of chondrocytes. J Biol Chem 279: 1747–1756. 14593093 10.1074/jbc.M310384200

[45] Wang X, Goh CH, Li B (2007) p38 mitogen-activated protein kinase regulates osteoblast differentiation through osterix. Endocrinology 148: 1629–1637. 17185377 10.1210/en.2006-1000

[46] Oh CD, Chang SH, Yoon YM, Lee SJ, Lee YS, et al (2000) Opposing role of mitogen-activated protein kinase subtypes, erk-1/2 and p38, in the regulation of chondrogenesis of mesenchymes. J Biol Chem 275: 5613–5619. 10681543 10.1074/jbc.275.8.5613

